# Bacterial and Host Determinants of Group B Streptococcal Infection of the Neonate and Infant

**DOI:** 10.3389/fmicb.2022.820365

**Published:** 2022-02-21

**Authors:** Anna Furuta, Alyssa Brokaw, Gygeria Manuel, Matthew Dacanay, Lauren Marcell, Ravin Seepersaud, Lakshmi Rajagopal, Kristina Adams Waldorf

**Affiliations:** ^1^Center for Global Infectious Disease Research, Seattle Children’s Research Institute, Seattle, WA, United States; ^2^Department of Global Health, University of Washington, Seattle, WA, United States; ^3^Morehouse School of Medicine, Atlanta, GA, United States; ^4^Department of Obstetrics & Gynecology, University of Washington, Seattle, WA, United States; ^5^Department of Pediatrics, University of Washington, Seattle, WA, United States; ^6^Department of Obstetrics and Gynecology, Sahlgrenska Academy, University of Gothenburg, Gothenburg, Sweden

**Keywords:** group B streptococcus, GBS, neonate, sepsis, meningitis

## Abstract

Group B streptococci (GBS) are Gram-positive β-hemolytic bacteria that can cause serious and life-threatening infections in neonates manifesting as sepsis, pneumonia, meningitis, osteomyelitis, and/or septic arthritis. Invasive GBS infections in neonates in the first week of life are referred to as early-onset disease (EOD) and thought to be acquired by the fetus through exposure to GBS *in utero* or to vaginal fluids during birth. Late-onset disease (LOD) refers to invasive GBS infections between 7 and 89 days of life. LOD transmission routes are incompletely understood, but may include breast milk, household contacts, nosocomial, or community sources. Invasive GBS infections and particularly meningitis may result in significant neurodevelopmental injury and long-term disability that persists into childhood and adulthood. Globally, EOD and LOD occur in more than 300,000 neonates and infants annually, resulting in 90,000 infant deaths and leaving more than 10,000 infants with a lifelong disability. In this review, we discuss the clinical impact of invasive GBS neonatal infections and then summarize virulence and host factors that allow the bacteria to exploit the developing neonatal immune system and target organs. Specifically, we consider the mechanisms known to enable GBS invasion into the neonatal lung, blood vessels and brain. Understanding mechanisms of GBS invasion and pathogenesis relevant to infections in the neonate and infant may inform the development of therapeutics to prevent or mitigate injury, as well as improve risk stratification.

## Introduction

**FIGURE 1:**
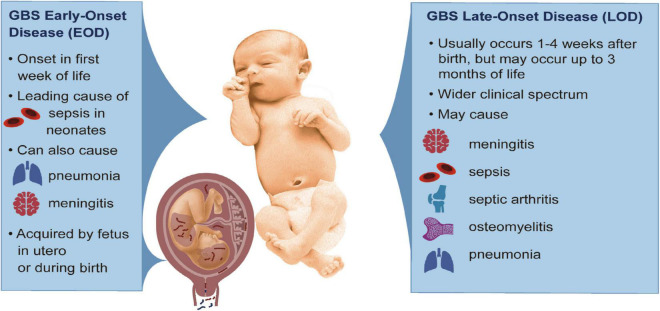
Clinical features of GBS EOD and LOD. The left panel describes characteristics of GBS EOD that arise from acquisition of GBS *in utero* or during childbirth. The right panel summarizes key features of GBS LOD acquired after birth and includes a wider clinical spectrum of disease. This baby was taken from Creative Commons: https://commons.wikimedia.org/wiki/File:Human-Male-Newborn-Infant-Baby.jpg.

Group B streptococci (GBS; *Streptococcus agalactiae*) are Gram-positive, β-hemolytic bacteria, which represent an important cause of neonatal and infant morbidity and mortality. GBS are typically commensal organisms and colonize the gastrointestinal (GI) and female lower reproductive tracts in approximately 20–25% of women (Russell et al., 2017a; Seale et al., 2017a). Although its presence in these niches is generally asymptomatic, during pregnancy GBS can ascend from the lower genital tract into the uterus and infect the fetus (Brokaw et al., 2021). An ascending GBS infection can cause invasive disease in the fetus, which is associated with a heightened risk for adverse pregnancy outcomes including preterm birth (PTB), stillbirth, and injury to fetal organs (Santi et al., 2007; Verani et al., 2010; Adams Waldorf et al., 2011; McAdams et al., 2012; Ku et al., 2015; Bianchi-Jassir et al., 2017; Seale et al., 2017b; Berardi et al., 2019; Ying et al., 2019; Brokaw et al., 2021; Horváth-Puhó et al., 2021; McCartney et al., 2021). Early-onset disease (EOD) refers to invasive GBS disease presenting in the first week of life and are thought to result from acquisition of an invasive GBS infection either in utero or during birth through exposure to vaginal fluids; typically, these cases present with sepsis, but may also include pneumonia and/or meningitis in severe cases (Figure 1). However, a majority of EOD cases occur within the first 24–48 h of life (Heath and Jardine, 2014; Nanduri et al., 2019), leading some to define cases of EOD as those manifesting within the first 72 h after birth (Lin et al., 2011; Berardi et al., 2019). When an invasive GBS infection develops between 7 and 89 days after birth, this is called late-onset disease (LOD, Figure 1). Transmission routes underlying LOD are more complex and have been linked to various sources. For example, there are limited case studies detecting GBS transmission to the neonate through breast milk (Elling et al., 2014; Buser et al., 2017; Tazi et al., 2019). Nosocomial transmission has been known to occur for babies born to GBS-negative mothers (Kim et al., 2006). Community transmission of GBS can also occur in formula-fed infants (Anthony et al., 1979; Morinis et al., 2011). In LOD, cases typically present with sepsis, but infections more frequently progress to meningitis; other clinical presentation of LOD include pneumonia, osteomyelitis, and/or septic arthritis. Globally, approximately 205,000 neonates are diagnosed with EOD and 114,000 with LOD each year (Bianchi-Jassir et al., 2017).

The pathogenesis of invasive GBS infections in neonates and infants is complex and there is no single approach to prevent all cases of PTBs, EOD and LOD. A multifactorial approach, such as one combining screening, antibiotics, and vaccination, will be needed to successfully reduce GBS invasive disease and adverse outcomes in neonates. To design effective therapeutics, more knowledge is needed to define how the use of GBS virulence factors impacts neonatal transmission routes and dissemination. We have previously described the bacterial and host determinants of GBS invasive disease in pregnancy by tracing the ascent of GBS from a colonized vaginal tract into the uterus and across the placenta (Brokaw et al., 2021). In this review, we focus on GBS infections of the neonate summarizing the epidemiology, sequelae of invasive disease and routes of GBS dissemination within the infant. We also define GBS virulence factors that enable dissemination to neonatal and infant target organs and potentiate disease.

## Methods

### Search Strategy and Selection Criteria

To identify recent and seminal GBS research relating to neonatal GBS invasive disease, including EOD and LOD, literature searches were performed using Google Scholar and PubMed Central. Variants of the terms “neonate,” “infant,” “early onset disease,” “late onset disease,” “antibiotic resistance,” “sepsis,” “pneumonia,” “meningitis,” “neurodevelopmental impairment,” “gut colonization,” “brain,” “lungs,” and “blood,” were used in combination with “Group B Streptococcus” or “*Streptococcus agalactiae*.” The literature search was limited to articles published between 2014 and 2021. Snowball searches were also used from reference lists of articles that met our search criteria. For research focused on GBS pathogenesis and virulence, studies were excluded if their methods and infection models were not particularly relevant for neonatal GBS infection.

## Clinical Outcomes and Risk Factors Associated With Early-Onset Disease and Late-Onset Disease

### Intrapartum Antibiotic Prophylaxis to Reduce Rates of Early-Onset Disease

Clinical practices markedly alter the outcome of invasive GBS disease in neonates. In the 1970s, GBS infections appeared as the leading cause of neonatal mortality and morbidity, igniting the need for potential therapeutics. During this time, EOD incidence was strikingly higher than LOD at 2–3 cases per 1,000 births and 0.2 cases per 1,000 birth, respectively (Schrag and Verani, 2013). The use of intrapartum intravenous ampicillin or penicillin in mothers were implemented in clinical trials and identified as highly effective at preventing EOD GBS (Boyer and Gotoff, 1986; Tuppurainen and Hallman, 1989; Garland and Fliegner, 1991; Schrag and Verani, 2013; ACOG, 2020). Guidelines for the use of intrapartum antibiotic prophylaxis (IAP) have been revised over the years to better mitigate risk of invasive GBS for pregnant individuals and their babies. There is no standard global approach for allocation of IAP and countries differ as to whether IAP is administered when clinical risk factors develop (i.e., preterm labor, prolonged rupture of membranes, and history of prior GBS invasive disease) or based on the results of universal screening with rectovaginal GBS cultures performed in all pregnancies. The adoption of universal GBS screening in late pregnancy and IAP, which occurred in the United States in 2002, became the most significant clinical practice to reduce neonatal invasive GBS disease. IAP decreased the incidence of GBS EOD from 1.7 cases per 1,000 live births to 0.22 cases per 1,000 live births; in contrast, the rate of LOD has not changed (Russell et al., 2017b; Berardi et al., 2019; Yeo et al., 2019). The inability of IAP to prevent *in utero* GBS transmission (that often leads to PTBs and stillbirths) or reduce LOD case rates has led to exploration of other clinical practices to prevent invasive GBS infections. Further, there is a strong push to develop novel therapeutic and prophylactic strategies that might more effectively target the wide range of GBS-associated outcomes.

Screening and IAP policies vary by country and have fluctuating effectiveness depending on the regional prevalence of GBS maternal colonization and distribution of serotypes. While universal screening programs test all pregnant women and treat all women who are rectovaginally colonized with GBS, a risk-based IAP policy only administers IAP if women are at higher risk for invasive disease (i.e., prolonged rupture of membranes and preterm labor) or if they have experienced adverse outcomes due to GBS in a previous pregnancy (Le Doare et al., 2017b). Universal screening is more successful at identifying GBS cases, but costlier to implement. In the United States, the American College of Obstetricians and Gynecologist has recommended universal screening for GBS between 36 0/7 and 37 6/7 weeks gestation (ACOG, 2020). In contrast, many regions with a relatively low burden of GBS disease have chosen a risk-based approach to IAP, like Sweden (Kolkman et al., 2013; Håkansson et al., 2017). However, a significant portion of GBS-associated adverse outcomes occur in low- and middle-income countries that may not have sufficient resources to implement IAP or diagnose neonates with GBS invasive disease (Le Doare et al., 2017b; Seale et al., 2017a).

### Group B Streptococci Typing and Antibiotic Resistance

GBS encodes a plethora of virulence factors that impact its ability to transition from asymptomatic colonizer to successful invasive pathogen. One major factor is the GBS sialylated capsular polysaccharide (CPS), which aids in evasion of host immunity (Carlin et al., 2009a,b; Gendrin et al., 2018). There are 10 major CPS types that inform GBS serotype classifications (Ia, Ib, and II–IX). Notably, six serotypes (I–V) account for 98% of isolates colonizing pregnant people and 99% of EOD and LOD cases (Madrid et al., 2017; Russell et al., 2017a; Nanduri et al., 2019). Serotype III is a major contributor to the burden of neonatal disease accounting for 62% of fetal and neonatal disease cases (Hall et al., 2017; Madrid et al., 2017). After serotype III, the most common GBS serotypes associated with EOD and LOD are Ia, Ib, and V (Madrid et al., 2017). In more recent studies interrogating invasive disease or GBS exposure in infants and neonates, proportions of EOD versus LOD varied greatly in each study (Table 1). Most of the invasive isolates observed in these cohorts belong to serotype III (range 40.7–77.9%), with the next most common belonging to serotypes Ia/b or V (Kang et al., 2017; Martins et al., 2017; Guan et al., 2018; Guo et al., 2018; Kao et al., 2019; Li et al., 2019; Wu et al., 2019; Plainvert et al., 2020; Awwad et al., 2021; Kekic et al., 2021; Lohrmann et al., 2021). Interestingly, serotype Ia (55.6%) and V (83.1%) were most common among the American and Icelandic infants, respectively, although in both cases serotype III was second (Björnsdóttir et al., 2019; Nanduri et al., 2019). Overall, these serotype profiles are consistent with earlier meta-analyses (Madrid et al., 2017).

**TABLE 1:** Summary of recent studies on antibiotic resistance in neonatal GBS isolates.

Location	Study design	Manifestations	Serotype	Resistance
China	Guan et al. (2018) Age: <3 m.o. (months old) Duration: 2011–2014	*N* = 70 EOD (70%) LOD (30%)	Ia (2.9%) Ib (14.7%) III (77.9%) V (4.4%)	Penicillin (0%) Ceftriaxone (0%) Vancomycin (0%) Linezolid (0%) Sulfamethoxazole (2.9%) Ofloxacin (5.9%) Clindamycin (51.5%) Erythromycin (57.4%) Tetracycline (95.6%)
	Guo et al. (2018) Age: newborns Duration: July–November 2015	*N* = 24 Sepsis (8.3%) Pneumonia (0%)	Ia (33.3%) Ib (25%) III (41.7%)	Penicillin (0%) Ceftriaxone (0%) Linezolid (0%) Vancomycin (0%) Chloromycetin (8.3%) Levofloxacin (37.5%) Clindamycin (58.3%) Tetracycline (70.8%) Erythromycin (70.8%)
	Li et al. (2019) Age: <3 m.o. Duration: 2013–2016	*N* = 93 EOD (36.5%) LOD (63.4%)	Ia (79.6%) Ib (12.9%) II (4.3%) V (3.2%)	Erythromycin (60.2%) Clindamycin (65.6%) Tetracycline (93.5%)
	Wu et al. (2019) Age: infants Duration: 2008–2015	*N* = 41 EOD (29.3%) LOD (41.5%)	Ia (7.3%) Ib (24.4%) III (63.4%) V (2.4%) NT (2.4%)	Penicillin (0%) Vancomycin (0%) Levofloxacin (19.5%) Clindamycin (82.9%) Erythromycin (87.8%) Tetracycline (95.1%)
France	Plainvert et al. (2020) Age: neonates Duration: 2007–2019	*N* = 1,262 EOD (31%) Bacteremia (75.6%) Meningitis (24.1%) LOD (69%) Bacteremia (50.9%) Meningitis (45.7%)	Ia (13.3%) Ib (2.8%) II (3.4%) III (74.2%) IV (1.2%) V (4.3%)	Penicillin (0%) Amoxicillin (0%) Vancomycin (0%) Gentamicin (0.2%) Amikacin (7.4%^∧^) Erythromycin (21%^∧^) Tetracycline (91%)
Portugal	Martins et al. (2017) Age: <1 y.o. (years old) Duration: 2005–2015	*N* = 218 EOD (51.8%) LOD^#^ (48.2%)	Ia (22%) Ib (5.5%) II (4.1%) III (58.7%) IV (1.4%) V (4.1%) VI (0.5%) VIII (0.5%) IX (0.5%) NT (2.8%)	Penicillin (0%) Vancomycin (0%) Levofloxacin (0%) Gentamicin (0.5%) Chloramphenicol (1.4%) Streptomycin (3.2%) Clindamycin (14.2%) Erythromycin (16.1%) Tetracycline (85.8%)
United States	Nanduri et al. (2019) Age: <3 m.o. Duration: 2006–2015	2,664 cases EOD (47.9%) Preterm birth (27.5%) Bacteremia (83.1%) Meningitis (9.5%) Pneumonia (6%) Septic shock (1%) Death (6.9%) LOD (52.1%) Preterm birth (41.8%) Bacteremia (61.1%) Meningitis (31.4%) Pneumonia (1.9%) Septic shock (2.8%) Death (5.4%)	1,743 isolates* Ia (55.6%) Ib (7.5%) II (9.4%) III (41.5%) IV (6.1%) V (11.3%)	1,727 isolates* Penicillin (0%) Ampicillin (0%) Vancomycin (0%) Clindamycin (20.8%) Erythromycin (44.8%)
Iceland	Björnsdóttir et al. (2019) Age: <1 y.o. Duration: 1996–2015	118 cases EOD (55.1%) LOD (44.9%)	Ia (18.6%) Ib (8.5%) II (4.2%) III (40.7%) IV (8.5%) V (83.1%)	Penicillin (0%) Chloramphenicol (0%) Levofloxacin (0%) Vancomycin (0%) Gentamicin (0%) Clindamycin (1%) Streptomycin (2%) Erythromycin (9%) Tetracycline (81.6%)
Taiwan	Kao et al. (2019) Age: <1 y.o. Duration: 2003–2017	182 cases EOD (22.5%) LOD^#^ (77.5%)	Ia (18.1%) Ib (8.2%) II (1.6%) III (64.8%) V (3.3%) VI (3.8%)	Penicillin (0%) Ampicillin (0%) Vancomycin (0%) Cefotaxime (0%) Clindamycin (64.3%) Erythromycin (68.1%)
South Korea	Kang et al. (2017) Age: <1 y.o. Duration: 1996–2015	98 cases EOD (16.3%) LOD^#^ (83.7%)	Ia (15.3%) Ib (13.3%) II (1%) III (51%) V (18.4%) VI (1%)	Penicillin (0%) Erythromycin (42.9%)
Serbia	Kekic et al. (2021) Age: <18 y.o. Duration: 2015–2020	60 cases EOD (68.3%) LOD (31.7%)	Ia (10%) Ib (8.3%) II (5%) III (58.3%) IV (3.3%) V (16.7%)	Macrolides (25%) Clindamycin (25%) Erythromycin (30%) Tetracycline (81.7%)
Palestine	Awwad et al. (2021) Age: newborns and infants Duration: 2018–2020	11 cases	II (9.1%) III (45.5%) V (18.2%) NT (27.3%)	Amoxicillin (0%) Cefotaxime (9.1%) Vancomycin (9.1%) Levofloxacin (9.1%) Ceftriaxone (18.2%) Cefuroxime (27.3%) Clindamycin (27.3%)
Germany	Lohrmann et al. (2021) Age: <3 m.o. Duration: 2009–2010	164 cases EOD (36.6%) LOD^#^ (63.4%)	Ia (17.1%) Ib (3.7%) II (3%) III (68.3%) V (6.1%) VI (0.6%) IX (0.6%) NT (0.6%)	Clindamycin (15.2%) Erythromycin (26.2%)

*^∧^Average of annual rates throughout study duration.*

*^#^LOD defined as 8 days to 1 year.*

*Only seven surveillance sites collected GBS isolates. GBS serotype and resistance phenotypes was only analyzed from a subset of cases.

GBS strains are categorized by CPS serotype but may also be sub-grouped into sequence types or clonal complexes (ST or CC, respectively) by multi-locus sequence typing (Jones et al., 2003). Four major ST are emerging including ST-1, -17, -19, and -23. Of these, serotype III ST-17 strains are especially concerning given their disproportionately high associations with EOD, LOD, sepsis, and meningitis (Manning et al., 2009; Tazi et al., 2010). In a retrospective analysis of serotype and ST laboratory-based surveillance in 10 United States states, the Active Bacterial Core Network found that ST-17 accounted for 78% of EOD (49/63) and 87% of LOD (161/186) cases caused by serotype III (McGee et al., 2021). This tight correlation between ST-17 and invasive neonatal disease stems from the presence of multiple mobile genetic elements that harbor ST-17-specific virulence factors (Teatero et al., 2016), including the GBS surface adhesin HvgA that facilitates invasion of the neonatal brain (Tazi et al., 2010). Hypervirulent ST-17 GBS strains account for 90% of clinical isolates in meningitis cases (Musser et al., 1989; Brochet et al., 2006; Poyart et al., 2008; Ortiz et al., 2014). ST-17 and -19 strains are also more likely to persist postpartum even following the administration of IAP (Manning et al., 2008), which may be due to the high association of ST-17 strains with antibiotic resistance (Kao et al., 2019).

However, antibiotic resistance is not restricted to ST-17 or other emerging hypervirulent complexes. Reduced sensitivity to many antibiotics, including penicillin G, ampicillin, clindamycin, and vancomycin has been detected in GBS-colonized pregnant women in Kenya, China, Ethiopia, South Korea, Brazil, Poland, and the United States (Lu et al., 2014; Yoon et al., 2015; Mengist et al., 2017; Burcham et al., 2019; do Nascimento et al., 2019; Jisuvei et al., 2020; Kaminska et al., 2020). Erythromycin and clindamycin resistance have been observed in adult patients in a Taiwanese hospital (Tsai et al., 2019), while penicillin and multidrug-resistant GBS were noted at multiple hospitals in Japan (Nagano et al., 2012, 2019). Fluoroquinolone resistant isolates were detected among patients in a long-term care facility in New York (Wehbeh et al., 2005). These drug resistant GBS are highly relevant, as they may transmit to highly susceptible neonates through vertical, nosocomial, or community routes.

As expected, drug resistance has also been noted among numerous neonatal populations (Table 1). The drug resistance profiles associated with the neonatal isolates from these studies were quite variable, but resistance to clindamycin (range 14.2–82.9%), erythromycin (range 16.1–87.8%), and tetracycline (range 70.8–95.6%) were noted in nearly all populations. Antibiotics commonly prescribed for IAP remain penicillin and ampicillin (Le Doare et al., 2017b), which had little to no observable resistance in these studies. Some guidelines also call for cephalosporins, vancomycin, clindamycin, or azithromycin for IAP in women with low or high risk of penicillin allergy (Le Doare et al., 2017b; Zhu and Lin, 2021). Resistance to cephalosporins (ceftriaxone and cefuroxime) and vancomycin were observed in a small study of Palestinian newborns and infants (Awwad et al., 2021), while clindamycin resistance appears to be relatively common with consistently high rates in different geographical regions (Martins et al., 2017; Guan et al., 2018; Guo et al., 2018; Kao et al., 2019; Li et al., 2019; Nanduri et al., 2019; Wu et al., 2019; Awwad et al., 2021; Kekic et al., 2021; Lohrmann et al., 2021). These findings highlight the importance of antibiotic susceptibility tests in clinical settings particularly when penicillin cannot be prescribed.

Further, rates of resistance are highly variable between studies and thus it is difficult to determine GBS susceptibility profiles on a global scale. Although most GBS isolates, particularly those in neonates, remain sensitive to first-line beta-lactam antibiotics used for IAP, the detection of strains that are resistant to these and other drugs raise concerns regarding the long-term efficacy of IAP against invasive GBS in neonates. These data also emphasize a need for more consistent surveillance of antibiotic resistance for GBS isolates and for novel therapeutic strategies that can be used safely to prevent neonatal disease.

### Increased Risk for Group B Streptococci-Associated Mortality and Neurodevelopmental Injury in Human Neonates

Invasive GBS disease can lead to neonatal mortality or an increased risk for death early in life (Table 2). Pneumonia and sepsis, which are common in EOD, are life-threatening conditions that often require neonatal intensive care, which may not be available in resource poor settings (Russell et al., 2017b; O’Sullivan et al., 2019; Horváth-Puhó et al., 2021; Mynarek et al., 2021). Mortality is also markedly higher among preterm neonates with EOD compared to term neonates (19.2 versus 2.1%, respectively) (Nanduri et al., 2019). In LOD, meningitis is more common and may present with a broader spectrum of clinical manifestations including lethargy, seizures, and abnormal body movements and stiffness (Dangor et al., 2015; Masroori et al., 2020). Notably, meningitis imparts a greater risk of mortality in early life. A retrospective cohort study of 2,258 children from Denmark and the Netherlands found that children with GBS meningitis (either EOD or LOD) had a higher 5-year mortality adjusted hazard ratio (aHR) than children without a history of GBS (Denmark: aHR 4.1, 95% CI 1.8–9.4; Netherlands: aHR 6.7, 95% CI 3.8–12.1) (Horváth-Puhó et al., 2021). Another population-based cohort study (*N* = 1,206) in Australia revealed that children with invasive GBS infections had three times the adjusted odds ratio (aOR) for death compared to all live births (aOR 3.0, 95% CI 2.1–4.3) (Yeo et al., 2019). GBS disease of the neonate, and especially meningitis, imparts an elevated risk for 5-year mortality in the survivors (Yeo et al., 2019).

**TABLE 2:** Studies reporting rates of case fatality, mortality, and NDI for GBS EOD and LOD.

Study	Cases of EOD	Cases of LOD	Case fatality rate (%) or mortality rate* by country and by age	RR or OR of NDI (by age)
Horváth-Puhó et al., 2021	Denmark *N* = 1,327 Netherlands *N* = 445	Denmark *N* = 234 Netherlands *N* = 252	Denmark (0–89 days) 96.6* (65.0–12.81) Denmark (0–5 years) 5.6* (3.9–7.4) Netherlands (0–89 days) 319.4* (231.8–407.1) Netherlands (0–5 years) 19.4* (14.2–24.7)	Denmark RR 1.77 (95% CI 1.44–2.18) by 10 y.o. Netherlands RR 2.28 (95% CI 1.64–3.17) by 10 y.o.
Masroori et al., 2020	*N* = 28	*N* = 15	Oman (<3 months) 7.0% (3/43)	–
Nanduri et al., 2019	*N* = 1,277	*N* = 1,387	United States (<90 days) EOD 6.9% (88/1,277) LOD 5.4% (75/1,387)	–
Dangor et al., 2015	*N* = 66	*N* = 56	South Africa (<3 months) Overall 18.0% (22/122) EOD 22.7% (15/66) LOD 12.5% (7/56)	Univariate OR 39.81 (5.27–1,751.09) by 3 mo. old Univariate OR 35.24 (4.66–1,550.57) by 6 mo. old
Madrid et al., 2017	*N* = 3,664	*N* = 2,003	Global meta-analysis (<3 months) Overall 8.4% (6.6–10.2%) EOD 10% (7.0–12.0%) LOD 7.0% (4.0–9.0%)	–

*Mortality rates expressed in events per 1,000 child years. RR, relative risk; OR, odds ratio; mo., months; y.o., years old.

Invasive GBS infections in the neonate and infant are also associated with long-term sequelae related to brain injury. Meningitis is a major risk factor for neurodevelopmental impairment (NDI) with 1 in 5 survivors of infant GBS disease developing moderate to severe NDI over 18-months (Kohli-Lynch et al., 2017; Harden et al., 2022). In Denmark and the Netherlands, any history of invasive GBS was associated with greater rates of neurodevelopmental injury versus unexposed newborns, as well as a higher risk of moderate and severe disability requiring educational support (Horváth-Puhó et al., 2021). Moreover, preterm infants were at the highest risk of NDI (Horváth-Puhó et al., 2022). A cohort study of GBS meningitis survivors in the United States assessed children younger than 6 years using the Mullen Scales of Early Learning to assess language, motor and cognitive ability. A similar study in children from South India showed that survivors of invasive GBS infection had greater motor impairment compared to age-matched children who were not exposed to GBS (John et al., 2022). Impaired neurologic functioning was observed in 50% (9/18) of patients and 28% (5/18) experiencing severe impairment (Libster et al., 2012). In the same study, children ages 6–12 who had GBS meningitis as neonates were evaluated using the Wechsler Individual Achievement Tool-II to assess academic performance (i.e., reading and mathematics). In these children, 29% (7/24) were classified as having mild-to-moderate impairment while 8% (2/24) were categorized as severe. Impairments were classified as either cognitive (e.g., repeating a grade level and profound global developmental delay) or medical (e.g., hydrocephalus requiring ventriculoperitoneal shunting, seizures, blindness, and sensorineural hearing loss) (Libster et al., 2012). Neonatal invasive GBS, and especially meningitis, may result in profound neurocognitive and medical sequelae that can persist into childhood and adulthood. Additionally, invasive GBS infection in neonates has also been linked to emotional and behavioral problems, such as anxiety, attention, and behavioral problems in school aged GBS survivors (Chandna et al., 2022). Interestingly, a recent study observed increased risk for NDI among male Dutch and Danish children with a history of invasive GBS disease compared to female children (van Kassel et al., 2022), emphasizing that further work is needed to understand sex-specific differences in the long-term sequelae of GBS disease, such as NDI.

### Perinatal Group B Streptococci Exposure in Rodents Is Associated With Meningitis and Neurodevelopmental Impairment With Sex-Specific Differences in Outcomes

Recent studies have observed NDI in neonatal rodents exposed to GBS during pregnancy or neonatal life. Maternal exposure to inactivated GBS during pregnancy resulted in neutrophil infiltration and placental inflammation that was associated with growth restriction and neurodevelopmental deficits in the neonatal mouse brain (Allard et al., 2019a). Similar to observations of sex-specific differences in NDI after invasive GBS disease in humans, male offspring were more likely to experience cerebral palsy-like neuromotor impairments. This may be due to dysmyelination of the white matter and reduced microglial density, which were pathologic observations in both humans and mice (Allard et al., 2019a). Following GBS-exposure *in utero*, male offspring had elevated IL-1β levels in the blood compared to females (Allard et al., 2019b). IL-1 has been shown to induce neurotoxicity and altered myelination (Cai et al., 2004; Allan et al., 2005; Allard et al., 2019b), suggesting that the pro-inflammatory response to GBS infection can, on its own, impact neonatal brain development and function, and may do so in a sex-specific manner. In another study, pregnant rats systemically infected with live GBS experienced a more severe neutrophil-driven chorioamnionitis in the placentas of male versus female fetuses. Male offspring displayed early signs of autism spectrum disorder including changes in social interaction, communication, and sensory processing, as well as atypical development of neurons and axons (Allard et al., 2017). In contrast, female offspring perinatally exposed to inactivated GBS show signs of hyperactivity and reduced inhibition during and following puberty (Allard et al., 2018). The same group linked the GBS-induced autism-like phenotypes to elevated IL-1β and neutrophil-chemotactic CXCL1 in the male fetuses’ placentas compared to those from females (Allard et al., 2019b). This sexually dichotomous pattern of GBS-induced inflammation may stem from differential regulation of immune responses by sex hormones, as has been observed for IL-1Ra-mediated sensitivity of male rats to LPS-induced febrile responses (Ashdown et al., 2007). Similarly, males may be more susceptible to inflammation-associated adverse outcomes in response to GBS due to testosterone activation of androgen receptor-expressing immune cells, which is thought to increase between gestational day 19 and postnatal day 4 in rats (Csernus, 1986; Lai et al., 2012). These and other mechanisms of sexually dimorphic outcomes following invasive GBS exposure should be further interrogated.

One limitation of the above studies is that they do not recapitulate vertical transmission through an ascending route. Another more biologically relevant mouse model utilized vaginal GBS infection of pregnant dams shortly prior to labor onset, to interrogate the role of GBS perinatal infection in NDI (Andrade et al., 2018). In this murine model, mortality was increased in the pups exposed to neuroinvasive ST-17 GBS compared to a non-pigmented isogenic mutant; however, GBS was recovered from the brain regardless of strain and accompanied by meningitis-associated edema and hemorrhage. Although elevated cytokines were not observed, frequencies and activation of resident myeloid cells were increased in the GBS-infected brains. Surviving neonates displayed learning and memory impairments during adulthood, which may be explained by signs of astrogliosis and neuronal apoptosis in the neonatal hippocampus (Andrade et al., 2018). Rodent models may serve as useful tools for future studies that aim to characterize how GBS, as well as host factors, that are involved in vertical transmission and neonatal invasive disease may contribute to the development of NDI.

### Immune Compromise as a Risk Factor for Perinatal, Neonatal, and Infant Group B Streptococci Infections

Immune compromise is a known risk factor for invasive GBS infections in adults (e.g., older age and diabetes mellitus) (Francois Watkins et al., 2019; Collin et al., 2020) and is also a factor in perinatal and neonatal GBS disease. In a retrospective study of adults with invasive GBS in the United Kingdom, 17.7% (479/3,156) of cases were pregnancy related (Collin et al., 2020). While pregnancy does not represent an immunosuppressed state *per se*, there are immunologic changes that impair the maternal adaptive immune response at the maternal-fetal interface and in the periphery to favor tolerance of the fetus. Similarly, the fetus and neonate have a highly specialized adaptive immune system. Although immaturity of the fetal and neonatal immune system was originally hypothesized (Medawar and Medawar, 1953; Rendell et al., 2020) evidence now favors tolerogenic factors in changing T cell function that make the neonate vulnerable to invasive GBS infections (Rackaityte and Halkias, 2020). Furthermore, studies have identified a synergistic risk of invasive GBS neonatal infections when the pregnant mother was also immunocompromised by another condition. For example, the risk of acquiring LOD is elevated in infants born to HIV-positive pregnant women. In a study of 122 children with invasive GBS disease over a 1-year period in South Africa, incidence rates of EOD were similar between HIV-exposed and unexposed infants (1.1 and 1.5 per 1,000 live births); however, in HIV-exposed infants there was a 4.7-fold greater rate of LOD than in unexposed infants (95% CI: 2.2–9.7). The case fatality rate for EOD and LOD was high at 18.0% with a 13-fold higher aOR (Dangor et al., 2015). Maternal diabetes also represents a risk factor for GBS colonization in pregnancy (Schuchat et al., 1990). Thus, conditions that compromise maternal immunity can increase risk for GBS neonatal invasive disease.

In immunocompetent mothers, the placental transfer of GBS-specific antibodies is an important modulator of neonatal susceptibility. Maternal IgG transfer is most abundant during the third trimester; the last 4 weeks of pregnancy is the window during which most antibodies are transferred (Malek et al., 1996; Saji et al., 1999; Palmeira et al., 2012). High neonatal levels of maternally derived antibodies specific for GBS lower the risk of EOD and LOD (Lin et al., 2001, 2003, 2004). This reduction in GBS risk is dependent on gestational age at birth (Lin et al., 2001, 2003). Infants born after 34 weeks of gestation had GBS-specific IgG levels that were 80% of maternal levels and a lower risk of developing EOD (Lin et al., 2001). In contrast, GBS-specific serum IgG levels in infants born before 34 weeks were at 20% of maternal levels (Lin et al., 2001), explaining in part why PTB may predispose these neonates to GBS invasive disease (Lin et al., 2001, 2003).

Additionally, neonatal susceptibility to GBS disease can also be attributed to their propensity for immunosuppressive responses to bacterial infection. For instance, neonatal mice rapidly produce high levels of IL-10 in response to GBS, which in turn suppresses neutrophil trafficking to infected tissues and thus prevents bacterial clearance. When IL-10 production is blocked, neonates exhibited increased neutrophil recruitment and reduced bacterial burden, showing that these neonates were resistant to GBS sepsis (Madureira et al., 2011). In another study, IL-10 production was activated through TLR2 recognition of GBS. Abrogation of TLR2 or IL-10 signaling resulted in efficient neutrophil influx to infected tissues and subsequent bacterial clearance (Andrade et al., 2013). Further, human neonates receiving colostrum high in IL-10 were twice as likely to become GBS colonized (Le Doare et al., 2017a), indicating the biological relevance of this mechanism.

Neonates also have intrinsic immune deficiencies that prevent clearance of GBS and contribute to severe disease. Compared to adults, neonates have defective T helper 1 (Th1) responses to GBS. In adults, splenocytes produce type 1 pro-inflammatory cytokines [IFN-gamma (IFN-γ), TNF-alpha (TNF-α), and IL-6] in response to GBS infection and recruit T cells through the early release of chemokines, such as CXCL9, CXCL10, and CCL3. In turn, CD4+ T cells activate and differentiate into Th1 cells, which produce robust level of IFN-γ and facilitate bacterial clearance (Clarke et al., 2016). Neonates, however, have impaired IL-12 and IL-18 production, which result in a diminished type 1 inflammatory response and IFN-γ release. Neonatal mononuclear cells stimulated with recombinant IL-12 and IL-18 can enhance IFN-γ production (La Pine et al., 2003). Exogenous administration of IFN-γ increased neonatal survival and led to partial clearance of GBS in blood (Cusumano et al., 1996). An additional immune deficiency in neonates is the reduced pool of T helper 17 cells (Th17) (de Roock et al., 2013). Accordingly, cells from cord blood of full-term neonates had diminished IL-17 levels in response to live GBS compared to adults, which is likely the result of a lack of Th17 cells in newborn cord blood. Interestingly, addition of IL-17 led to an increase in IFN-γ production in neonates in response to GBS, suggesting a synergistic effect on the production of IL-17 and IFN-γ (Caron et al., 2014).

Although neonates have several neonatal immune perturbations that predispose to an invasive GBS infection, they can paradoxically also exhibit exaggerated inflammatory responses that promote disease. At a high bacterial burden, preterm infants had elevated IL-6 levels compared to adults, which is consistent with the inflammatory nature of neonatal GBS sepsis (Currie et al., 2011). Additionally, GBS-stimulated neutrophils can drive proinflammatory Th1 and Th17 characteristics in neonatal CD4+ regulatory T cells through cell–cell contact and soluble mediators. This bias to proinflammatory responses from canonically immunosuppressive adaptive immune populations, suggesting that GBS can curtail protective immunosuppressive responses to favor inflammatory responses that exacerbate GBS disease in neonates (Lin et al., 2018). Neonatal immune responses to GBS are multifactorial and thus, additional insights are needed on how neonatal immune deficiencies may simultaneously drive inflammation and dampen protective host defenses to increased susceptibility to invasive disease.

## Neonatal Group B Streptococci Invasive Disease of the Lung

**FIGURE 2:**
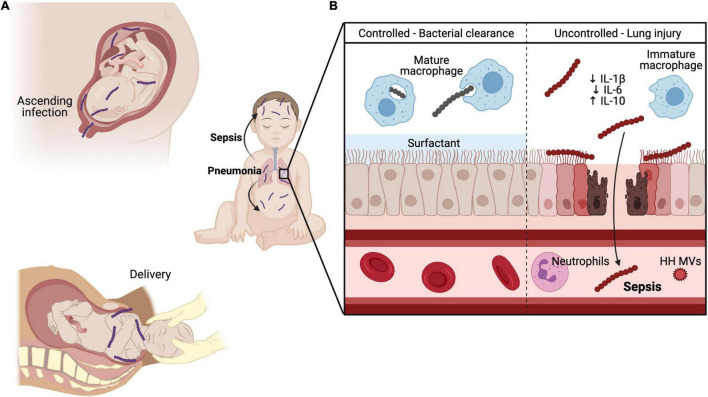
GBS pulmonary and systemic infection in neonates. GBS can be transmitted to the developing fetus **(A)** through ascending infection from the maternal vaginal tract and crossing of the maternal-fetal interface or through aspiration of infected vaginal fluid during delivery. **(B)** In healthy adult lungs, GBS is readily phagocytosed and killed by mature macrophages through Sn recognition of GBS CPS. Additionally, lung surfactant promotes efficient gas exchange and inhibits the action of hemolysin, thereby protecting and maintaining the lung barrier. Conversely, the developing neonate has several maturational deficiencies that contribute to susceptibility to invasive GBS infection. CPS engagement with Siglec-E blunts innate immune responses by immature alveolar macrophages through reduced IL-1β and IL-6 production and elevated IL-10 levels. Hemolysin penetrates lung epithelium and endothelium through direct cytolytic injury and promotes neutrophil recruitment to the lungs through IL-8 production. However, during systemic infection with a hyperhemolytic (HH) GBS, HH membrane vesicles (MVs) prevent oxidative killing of GBS by quenching reactive oxygen species (ROS), a major component of the oxidative burst. Created in part with BioRender.com.

The neonatal lung is a highly susceptible organ to GBS invasive disease. GBS can enter the fetal or neonatal lung with aspiration of GBS-infected amniotic fluid or vaginal fluids during delivery (Brokaw et al., 2021). This process deposits GBS directly into the lung, a mucosal barrier site that is poised to quickly respond to insults. Multiple mechanisms act to protect the lung from invading pathogens, including surfactants that entrap pathogens and mechanical clearance of pathogens by ciliated lung epithelial cells (Han and Mallampalli, 2015). GBS must evade these and other mechanisms to gain access to the lung mucosa. Next, GBS must adhere to the lung epithelium to invade the cell. After establishing infection in the lung, GBS can invade the lung microvascular endothelium to enter the bloodstream and cause sepsis (Figure 2). We describe how GBS induces lung disease in the neonate by considering in turn its ability to evade immune detection, adhere to pulmonary cells and breach several host barriers.

### The Group B Streptococci Capsule Confers Immune Evasion in the Neonatal Lung

To invade the neonatal lung, GBS must first evade host immune defenses. A major GBS factor enabling immune evasion is CPS (Rubens et al., 1987). GBS can express one of ten CPS, each with unique structural and antigenic features (Paoletti and Kasper, 2019). Despite structural and antigenic diversity, all CPS are terminally capped with a sialic acid (Wessels et al., 1989). Interestingly, this conserved sialic acid motif is also abundantly present on the surface of all mammalian cells. These host sialic acids bind to sialic acid-binding immunoglobulin-type lectins (Siglecs), a family of receptors that are expressed on the surface of innate and adaptive immune cell populations. Most Siglecs function as an inhibitory receptor that prevents aberrant immune activation (Crocker et al., 2007; Pillai et al., 2012). The heavily sialylated CPS on the GBS surface engages Siglec-E to blunt innate immune activation and suppress inflammatory responses in intranasally infected adult mice (Chang et al., 2014a). GBS takes advantage of this inhibitory mechanism to evade immune detection by the host (Carlin et al., 2007) through molecular mimicry of the host sialic acid. Despite these immune evasion strategies, the host can still induce bactericidal responses against GBS through a unique Siglec called sialoadhesin (Sn, also known as Siglec-1). Unlike other Siglecs, Sn recognizes sialylated GBS and subsequently stimulates inflammatory responses. Sn recognition of CPS has been shown to be critical for macrophage phagocytosis and efficient clearance of GBS in systemically infected adult mice. Additionally, this interaction facilitates crosstalk between the innate and adaptive immune responses because Sn recognition stimulates protective antibody responses against GBS (Chang et al., 2014b).

In neonates, however, the immune system fails to efficiently clear GBS. A study of the cellular and molecular mechanisms underlying neonatal susceptibility to GBS-associated lung injury used a mouse model and focused on an analysis of Siglec receptor and Sn expression alveolar macrophages (Lund et al., 2020). Lung-resident alveolar macrophages are highly specialized macrophages that survey the air-filled alveoli of the lungs and are critical for initiating pulmonary immune responses (Kopf et al., 2015). In the GBS mouse model of pneumonia, neonatal mice had higher rates of mortality and lung injury than juvenile or adult mice. While both adult and neonatal alveolar macrophages expressed Siglec-E, the underdeveloped neonatal alveolar macrophages had significantly reduced levels of Sn at the first day of life, as well as delayed expression of Sn during acute infection of the lungs. Thus, in neonates, CPS engages the inhibitory Siglec-E on alveolar macrophages effectively suppressing innate immune activation. In adults, CPS engages the inhibitory Siglec-E on mature alveolar macrophages, but also the activating Sn, which enables clearance of GBS (Lund et al., 2020). Therefore, GBS exploits the immaturity of the neonatal innate immune system and the delayed expression of Sn to prevent bacterial clearance and promote uncontrolled GBS infection in the lungs.

### Group B Streptococci Adhesion to Lung Epithelia and Subsequent Endothelial Invasion

After evading the first line of pulmonary immune defense, GBS must adhere to the pulmonary epithelium to successfully invade the lung. GBS employs a multitude of surface-associated adhesins to interact with the lung epithelium, including Spb1 (pili), BibA, and fibrinogen binding protein (FbsB) (Santi et al., 2007; Sharma et al., 2013; Korir et al., 2014; Zürn et al., 2020). In addition to the ability to bind components of the extracellular matrix, these adhesins also confer other functions such as cellular invasion and immune evasion (Rajagopal, 2009). Importantly, these adhesins poise GBS for invasion of host cells, which can promote dissemination into other vulnerable host compartments.

Once GBS has established a niche in the lung mucosa, it can penetrate lung endothelial barriers to gain access to the bloodstream. The loss of endothelial barrier integrity appears to be largely mediated through the actions of the GBS pore-forming toxin, hemolysin (also known as the β-hemolytic pigment, β-hemolysin/cytolysin, or granadaene) (Nizet et al., 1997; Whidbey et al., 2013). Previous studies have demonstrated that hemolysin is cytolytic to human alveolar epithelial cells, in addition to pulmonary arterial and lung microvascular endothelial cells (Nizet et al., 1996; Gibson et al., 1999). Hemolysin also stimulates the release of the neutrophil chemoattractant IL-8 and inflammatory cascades in lung epithelial cells, which further compromises barrier function (Doran et al., 2002). The hemolysin-mediated hyperinflammatory response and subsequent breakdown of lung architecture has also been corroborated in a biologically relevant vertical transmission model that recapitulates human maternal-fetal transmission (Andrade et al., 2018). Maternal colonization of pregnant mice with a hyperhemolytic GBS strain led to pups with a higher mortality rate, bacterial loads in multiple organs (liver and brain) and lung inflammation compared to an isogenic strain lacking *cylE*, which encodes the β-hemolytic pigment. The GBS hemolysin is a key virulence factor enabling invasion of the neonatal blood vessels and dissemination.

GBS hyperhemolytic membrane vesicles (MVs) have also been shown to exacerbate pulmonary and systemic GBS infection in neonatal mice. In a systemic challenge model, co-infection of neonatal mice with hemolytic MVs and nonhemolytic GBS resulted in reduced survival compared to infections with nonhemolytic GBS and/or nonhemolytic MVs alone (Armistead et al., 2021). Co-infection with hemolytic MVs was also associated with increased mortality and increased neutrophil recruitment to the lungs and greater lung inflammation. Hemolytic MVs have also been shown to inhibit the activity of reactive oxygen species (ROS), suggesting that GBS may resist neutrophil oxidative killing by quenching ROS (Armistead et al., 2021). MVs composition and abundance are demonstrated to vary based on serotype and lineage indicating differential impact on promoting virulence and systemic infection (McCutcheon et al., 2021). In addition to MVs, other GBS virulence factors may also contribute to lung injury and blood vessel invasion. GBS expresses an extracellular DNase, called NucA, which degrades the DNA matrix within neutrophil extracellular traps (NET) (Derré-Bobillot et al., 2013). In an intranasal challenge model, NucA was required for GBS persistence in the lungs and blood, suggesting that NucA may play a role in GBS lung injury and dissemination in the bloodstream (Derré-Bobillot et al., 2013). In summary, several virulence factors act in concert to promote lung injury and blood vessel invasion.

#### Animal Models of Group B Streptococci Fetal Lung Injury

**FIGURE 3:**
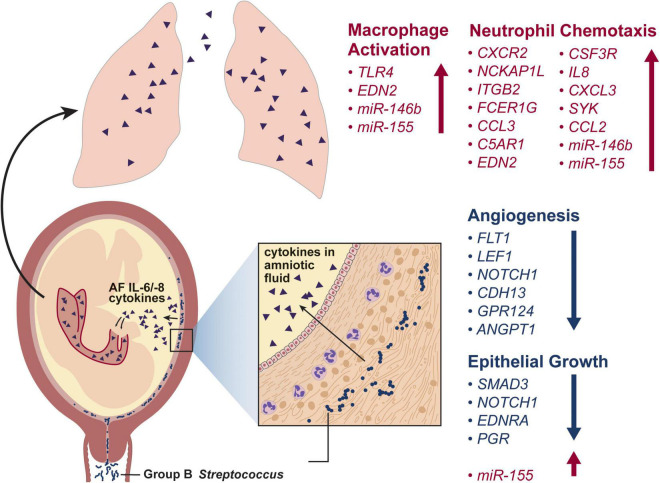
This conceptual model illustrates the progression of a GBS infection from the vagina and into the choriodecidual space. Here, GBS induces release of inflammatory cytokines, which traffic into the amniotic fluid and are inspired by the fetus. Exposure of the fetal lungs to inflammatory cytokines induces macrophage activation, neutrophil chemotaxis and inhibits angiogenesis and epithelial growth. The differentially regulated genes shown in the fetal lung after a GBS-associated inflammatory injury are based on two studies in a nonhuman primate model (Adams Waldorf et al., 2011; McAdams et al., 2015).

Fulminant infections in neonates may be due to acquisition of GBS *in utero.* Nonhuman primate (NHP) models have advanced the understanding of *in utero* infections that lead to GBS invasion and fetal lung injury, which can manifest as EOD. In a pigtail macaque (*Macaca nemestrina*) model, GBS is inoculated into the choriodecidual space between the uterine muscle and the placental chorioamniotic membranes (Adams Waldorf et al., 2011; Boldenow et al., 2016; Coleman et al., 2021); the choriodecidual space is hypothesized to be the site of first contact with the placenta after GBS ascension from the lower genital tract. Once in the choriodecidual space, the outcome of the infection – either successful eradication by the host immune response or a failure to control the infection – is largely dependent upon the virulence factors expressed by the GBS strain (Adams Waldorf et al., 2011; Boldenow et al., 2016; Coleman et al., 2021). However, even when the infection is eradicated, proinflammatory cytokines produced by the host immune response in the chorioamniotic membranes can traffic into the amniotic fluid and induce fetal lung injury (Adams Waldorf et al., 2011). Further, cytokine injury of the fetal lung can occur silently in the presence or absence of preterm labor or chorioamnionitis (neutrophilic infiltration of the membranes) (Adams Waldorf et al., 2011). When fetal lung tissue from this model was analyzed by microarray, many gene sets critical for lung development were downregulated including angiogenesis, morphogenesis, and cellular differentiation gene sets (McAdams et al., 2012, 2015; Figure 3). These findings suggest that GBS-induced inflammation *in utero* can promote fetal lung injury in the absence of bacterial invasion and present as pneumonia in the first week of life.

Another key finding from NHP models of GBS invasive disease in pregnancy is that inhibition of the chemokine response may prevent preterm labor, but not progression of fetal lung injury (Coleman et al., 2020). In a pregnant pigtail macaque model, prophylaxis with a broad-spectrum chemokine inhibitor (BSCI) prior to choriodecidual inoculation of GBS completely suppressed preterm labor (Coleman et al., 2020). Cesarean delivery and fetal necropsy were performed 3 days after GBS inoculation in the BSCI group. Although the uterus remained quiescent with BSCI treatment, rates of fetal pneumonia and GBS invasion of the amniotic cavity was higher after BSCI treatment compared to GBS alone (Coleman et al., 2020). Immunotherapeutic development for preterm labor or fetal lung injury should occur with consideration of antibiotic therapy and a careful evaluation for amniotic fluid microbes.

A neonatal rabbit model of GBS lung injury has been used to test the role of exogenous lung surfactant as a therapeutic in ameliorating GBS disease (Xu et al., 2019). Lung surfactants function to decrease the alveolar surface tension and thereby, improve gas exchange and protect the alveoli from hyperoxic stress (Herting et al., 1997). Interestingly, the hemolytic activity and subsequent pro-inflammatory effects of hemolysin is inhibited by a major component of lung surfactant, dipalmitoylphosphatidylcholine (DPPC) (Nizet et al., 1996). However, premature neonates and very low birth weight neonates are DPPC-deficient, which may contribute to their increased risk for mortality due to invasive GBS disease (Liu et al., 2004). Treatment with surfactant in an intratracheal rabbit model suppressed GBS proliferation in the lungs and progression to systemic infection, alleviated proinflammatory responses and improved lung mechanisms in near-term neonatal rabbits (Xu et al., 2019). This data suggests that there may be a role for exogenous surfactant to mitigate GBS-induced neonatal lung injury.

## Neonatal Group B Streptococci Invasive Disease of the Intestine and Brain

**FIGURE 4:**
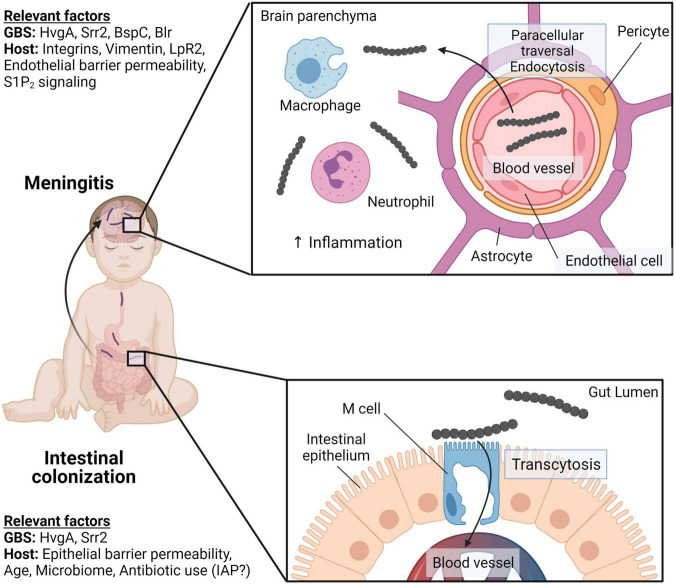
GBS intestinal colonization and meningitis in neonates. Neonates can acquire GBS through vertical transmission or community sources resulting in intestinal colonization. Both GBS proteins, HvgA and Srr2, facilitate adhesion to the intestinal epithelium. However, only Srr2 engages with M cells in Peyer’s patches to facilitate GBS crossing of the intestinal barriers through M cell transcytosis. GBS can gain access to the brain through the bloodstream and crossing the blood–brain barrier (BBB). Srr2 interacts with integrins that are overexpressed in the postnatal brain to adhere to brain endothelial cells. GBS enters endothelial cells *via* endocytosis and can induce endothelial-mesenchymal transition to facilitate BBB disruption. GBS BspC binds to host vimentin and stimulates phagocyte recruitment and brain inflammation. Created with BioRender.com.

Neonatal swallowing of vaginal fluid during childbirth or breast milk has been proposed as an entry point for GBS that may lead to colonization of the neonatal intestine and bacterial dissemination into the bloodstream (Kotiw et al., 2003; Zimmermann et al., 2017; Nicolini et al., 2018). It is thought that GBS can colonize the colon and potentially the small intestine, eventually leading to bacterial invasion of the intestinal epithelium and access to the bloodstream. GBS can then cross the blood–brain barrier (BBB) to infect the neonatal brain and cause neurological injury in the neonate (Figure 4; Landwehr-Kenzel and Henneke, 2014). Like GBS-associated lung injury, the pathogenesis of GBS invasive disease in the intestine is reliant on the pathogen’s ability to overcome several host barriers to gain access to vulnerable host niches.

### Group B Streptococci Intestinal Colonization and Traversal Across the Intestinal Wall

As has been suggested for other mucosal sites, such as the maternal vaginal tract, the composition of the neonatal intestinal microbiota may affect susceptibility to GBS. A recent murine study has shown that neonatal mice were more susceptible to oral GBS challenge at postnatal day 1 (P1) compared to postnatal day 5 (P5); taxonomic analysis of the intestinal microbiome at these timepoints observed an underdeveloped phenotype at P1 compared to P5. Reconstitution of P1 pups with P5 microbiota rescued this phenotype, suggesting that susceptibility to GBS intestinal colonization is microbiome-dependent (Travier et al., 2021). However, it appears that microbiome-independent intestinal epithelial Wnt signaling is involved in the dysregulation of epithelial polarity and loss of cell-to-cell junctions that ultimately favors GBS translocation across the gut epithelium. Neonates that experienced intestinal barrier disruption were predisposed to GBS dissemination, bacteremia, and subsequent neuroinvasion (Travier et al., 2021). Further studies are needed to corroborate the key intestinal taxa and mechanisms that drive differences in gut colonization susceptibility, as well as to determine whether these factors may be relevant in humans. In addition, a limitation is that oral challenge of specific pathogen-free mice with GBS does not adequately recapitulate transmission to neonates that may occur during delivery or *via* infected breast milk.

Human clinical studies also indicate that intestinal GBS may promote the development of meningitis. A longitudinal study tracking microbial colonization of the gut observed that, prior to LOD-associated sepsis, GBS clones found in the blood of septic preterm infants was genetically identical to GBS clones previously isolated from the infant’s stool (Carl et al., 2014). To colonize the gut, GBS utilizes adhesins to adhere to the intestinal epithelium. Hypervirulent GBS adhesin (HvgA), a surface protein conserved among hyperinvasive ST-17 isolates, is a vital adhesin for intestinal colonization that mediates binding to intestinal epithelial cells. Heterologous expression of HvgA in non-adhesive GBS strains conferred an adhesive phenotype. Oral challenge of mice with HvgA-expressing GBS results in invasion into cecal tissue, suggesting an additional role for HvgA in translocation of the intestinal barrier (Tazi et al., 2010).

Another ST-17 surface protein, Srr2, is also implicated in binding to the intestinal wall. Srr2 interacts with intestinal M cells to enhance GBS transcytosis across the intestinal epithelium, permitting subsequent access to the bloodstream (Hays et al., 2019). GBS transcytosis across the intestinal barrier is dependent on the differentiation of the M cell. Throughout pregnancy, neonates are exposed to very high levels of pregnancy-related hormones, including estradiol and progesterone. These hormones promote the maturation of M cells and thus potentiate Srr2-mediated GBS traversal across the intestinal barrier and ST-17 GBS invasiveness (Hays et al., 2019). Srr2 has also been shown to bind fibrinogen and plasminogen with high affinity, resulting in the initiation of bacterial aggregates that are engulfed by macrophages (Seo et al., 2013). Finally, Srr2 improves GBS intracellular survival within macrophages and is involved in mediating persistence in the blood (Six et al., 2015), suggesting that hyperinvasive GBS can subvert the innate host immune response to prevent clearance from the bloodstream. In summary, the intestine is an important entry point for GBS, and bacterial invasion of the neonatal brain and bloodstream is mediated by expression of key virulence factors, like HvgA and Srr2.

### Group B Streptococci Sepsis and Brain Microvasculature Adhesion Facilitates Blood–Brain Barrier Invasion

Many virulence factors that are critical for colonization and invasion of the neonatal intestine also facilitate BBB traversal. For example, HvgA facilitates adhesion and crossing of the choroid plexus and microvascular endothelial cells that constitute the BBB. Oral challenge of neonatal mice with HvgA-expressing GBS leads to bacterial invasion of the brain and meningitis (Tazi et al., 2010). However, animal models of GBS sepsis using an intravenous route of infection can also lead to meningitis. Interestingly, a bloodstream infection by HvgA-deficient GBS replicated well in the blood, but BBB invasion was impaired (Tazi et al., 2010). Like HvgA, Srr2 plays a dual role in intestinal colonization and BBB invasion; however, unlike HvgA, Srr2 binds to α5β1 and αvβ3 integrins to mediate adhesion and internalization by cells in the BBB. These integrins are overexpressed in the postnatal brain, contributing to the susceptibility of neonates to GBS-associated meningitis (Deshayes de Cambronne et al., 2021). Blocking this interaction hindered GBS translocation across the BBB, demonstrating that Srr2 is a potential vaccine target to prevent meningitis (Deshayes de Cambronne et al., 2021). Lastly, the BspC adhesin is important for *in vitro* adhesion of GBS to brain endothelial cells through the cytoskeletal protein vimentin; mice infected with BspC-deficient GBS exhibit a lower bacterial burden in the brain, inflammation, and mortality (Deng et al., 2019). Some GBS virulence factors that enable penetration of the neonatal intestine also facilitate traversal of the BBB.

### Group B Streptococci Virulence Factors Exploit Host Signaling Pathways to Penetrate the Blood–Brain Barrier

The BBB is a tightly controlled interface that separates the microvasculature from the parenchymal tissue of the brain. This protects the central nervous system from circulating pathogens that may be contained in the blood. This essential barrier is made up of interlinked layers of perivascular pericytes and astrocytes that sit atop a basal membrane made of the extracellular matrix, and each component is involved in regulating BBB integrity (Obermeier et al., 2013). Unique structural features, such as apical tight junctions that prevent paracellular passage and highly selective transport systems, contribute to barrier functions that pathogens must overcome to gain access to the brain (Al-Obaidi and Desa, 2018). GBS has several virulence factors within its arsenal that are capable of disrupting BBB function through several mechanisms, including bacterial invasion of BBB cells, direct cellular injury by bacterial toxins, or induction of proinflammatory cascades that compromise BBB integrity.

Multidimensional quantitative proteomics of infected whole brains revealed that GBS infection altered proteomic signatures corresponding to upregulated interferon signaling and leukocyte recruitment. In the brain microvasculature, proteins involved in barrier function, such as maintaining integrity of the BBB after brain hemorrhage, were downregulated (Campeau et al., 2020). This loss of BBB integrity during GBS infection is also mediated through activation of the TLR2 pathway and subsequent induction of Snail1 signaling that leads to reduced expression of tight junction components (Kim et al., 2015). Interestingly, many signaling components that mediate loss of tight junctions in the BBB also result in the loss of vaginal and placental epithelial function *via* epithelial-mesenchymal transition (Vornhagen et al., 2018).

The immature intestinal epithelial barrier provides another example of how GBS exploits host signaling cascades to facilitate dissemination to the brain. Neonatal mice infected with GBS displayed dysregulation of the epithelial junction proteins E-cadherin, occludin, and claudin-3 (Travier et al., 2021). Whereas these proteins are concentrated apically in the properly polarized adult intestinal epithelium, they are more diffuse in neonates (Travier et al., 2021). This improper localization of E-cadherin in the neonatal intestinal epithelium was associated with elevated β-catenin, which is indicative of active Wnt signaling. In addition to its role in loss of epithelial polarity and reduced cell-to-cell junctions, transcripts associated with Wnt signaling were elevated in neonatal versus adult mice, suggesting that this pathway may play a role in the heightened susceptibility of neonates to GBS gut translocation and dissemination to the brain (Travier et al., 2021).

GBS can also exploit other host pathways to facilitate infection of the brain. In a novel *Drosophila* infection model, GBS Blr, a lipoprotein on the bacterial surface, interacts with host LpR2 to translocate into the brain through endocytosis. GBS engagement of LpR2 through Blr is required for GBS infiltration of the brain as corroborated in a mouse model of infection (Benmimoun et al., 2020). Additionally, a network-based targeted approach demonstrated that GBS modulates host S1P2 signaling to facilitate transcellular penetration of the BBB. Pharmacological inhibition of S1P2 improved GBS meningitis outcomes, highlighting a potential therapeutic for invasive GBS infection in the brain (Zhu et al., 2021). Altogether these studies show that GBS employs several unique mechanisms to exploit host signaling pathways to cross the BBB.

## Summary

Early-onset disease and LOD are invasive GBS infections of the neonate and infant that contribute to neonatal mortality and long-term disability. In this review, we considered how GBS exploits vulnerabilities in immune defenses to initiate and propagate infection within the neonatal lung, intestine and brain. The pathogenesis of GBS invasive disease is reliant on the organism’s ability to overcome host barriers. The use of animal models that closely mirror GBS maternal-infant transmission and pathogen acquisition by the infant has been pivotal in identifying how these bacteria exploit the underdeveloped neonatal immune system for dissemination and pathogenesis. GBS expresses a variety of virulence factors including CPS, hemolytic pigment, adhesins and extracellular enzymes that can act in concert to subvert, counteract, or promote immune responses conducive to GBS dissemination in the neonate. Many of these virulence factors are critical for colonization and/or invasion of GBS at various host niches leading to neonatal disease. While GBS screening coupled with IAP has been successful in reducing invasive disease, the emergence of antibiotic resistant GBS strains raises concerns about the long-term efficacy of this approach. Unravelling the mechanisms of GBS colonization, invasion and pathogenesis leading to EOD or LOD is key to discovering novel antimicrobial targets that will allow for the development of new therapeutics and prophylaxis strategies to better control and prevent invasive GBS disease in the neonate. Fully elucidating the molecular basis of interactions between GBS and the neonate will be essential to accomplish this endeavor.

## Author Contributions

All authors listed have made a substantial, direct, and intellectual contribution to the work, wrote the manuscript, and approved it for publication.

## Conflict of Interest

The authors declare that the research was conducted in the absence of any commercial or financial relationships that could be construed as a potential conflict of interest.

## Publisher’s Note

All claims expressed in this article are solely those of the authors and do not necessarily represent those of their affiliated organizations, or those of the publisher, the editors and the reviewers. Any product that may be evaluated in this article, or claim that may be made by its manufacturer, is not guaranteed or endorsed by the publisher.
